# Tumeur noire de la pyramide nasale

**DOI:** 10.11604/pamj.2014.19.82.4942

**Published:** 2014-09-25

**Authors:** Ahlam Abdou, Badredine Hassam

**Affiliations:** 1 Service de Dermatologie, CHU Ibn Sina, Université Med V, Souissi, Rabat, Maroc

**Keywords:** Tumeur noire, pyramide nasale, stroma fibro-inflammatoire, Black tumor, nasal pyramid, fibro-inflammatory stroma

## Image in medicine

Les tumeurs historiques (< 1% des carcinomes cutanés) sont des tumeurs géantes, de diamètre supérieur à 10 cm, et de longue durée d’évolution. Le CBC infiltrant est un carcinome mutilant de mauvais pronostic. Il représente 40% des carcinomes de la pointe et l'aile du nez. Les CBC de la pointe nasale, ont une histologie agressive confortant la chirurgie comme le traitement de choix. Le traitement consiste en une chirurgie de reconstruction avec un lambeau de transposition de type Limberg à pédicule supérieur, pris sur l'auvent nasal et la joue, ou une greffe de peau totale. La place de technique de chirurgie micrographique dans cette localisation mérite d’être soulignée. Nous rapportons l'observation de la patiente BM, 64 ans présentant une tumeur ulcéro-bourgeonnante du nez douloureuse, nauséabonde saignant au contact évoluant depuis 4 ans. L'examen clinique trouve une tumeur ulcéro-bourgeonnante croûteuse noirâtre perlée mesurant 10cm sur 6cm au dépend de la pyramide nasale mettant à nu la muqueuse nasale. La base est représentée par le plancher de la muqueuse nasale. L'histologie de la tumeur médio-faciale a objectivé un tissu ulcéré siège d'une prolifération carcinomateuse infiltrant le derme, les cellules basaloides munies de noyaux peu atypiques, un cytoplasme basophile et un stroma fibro-inflammatoire, en faveur d'un CBC infiltrant. Le bilan d'extension objective des adénopathies jugulaires droites d'allure inflammatoires, un processus tumoral infiltrant avec envahissement du tissu sous-cutané adjacent et lyse osseuse (os propre du nez et maxillaire supérieur). Une radiothérapie suivie d'une chirurgie de réparation par lambeau de rotation frontonasal ont été proposée.

**Figure 1:**
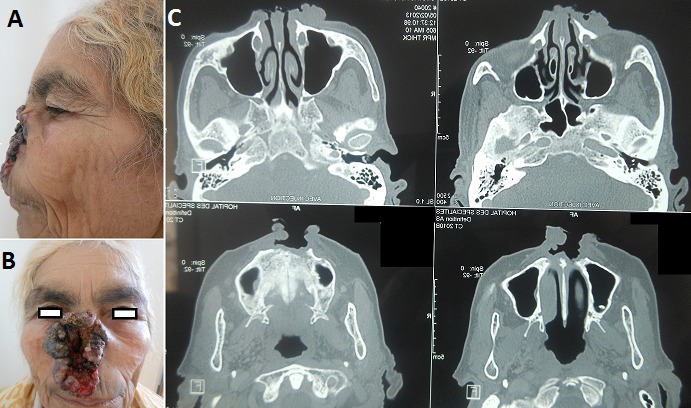
(A) tumeur noire centrofaciale ulcéro-bourgeonnante avec à nu de la muqueuse nasale; (B) Destruction de la pyramide nasale; (C) Lyse osseuse

